# Hibiscus Acid from *Hibiscus sabdariffa* L. Inhibits Flagellar Motility and Cell Invasion in *Salmonella enterica*

**DOI:** 10.3390/molecules27030655

**Published:** 2022-01-20

**Authors:** Ixchell Y. Sedillo-Torres, Álvaro O. Hernández-Rangel, Yolanda Gómez-y-Gómez, Daniel Cortés-Avalos, Blanca Estela García-Pérez, Juan C. Villalobos-Rocha, César H. Hernández-Rodríguez, Luis Gerardo Zepeda-Vallejo, Paulina Estrada-de los Santos, María Elena Vargas-Díaz, Jose Antonio Ibarra

**Affiliations:** 1 Departamento de Microbiología, Escuela Nacional de Ciencias Biológicas, Instituto Politécnico Nacional, Prol. de Carpio y Plan de Ayala S/N Col. Santo Tomás Alc. Miguel Hidalgo, Ciudad de México 11340, Mexico; iixchell.yureimy@gmail.com (I.Y.S.-T.); daniel.corav@gmail.com (D.C.-A.); abrilestela@hotmail.com (B.E.G.-P.); jc.villalobosrocha@yahoo.com (J.C.V.-R.); chdez38@hotmail.com (C.H.H.-R.); pestradadelossantos@gmail.com (P.E.-d.l.S.); 2 Departamento de Química Orgánica, Escuela Nacional de Ciencias Biológicas, Instituto Politécnico Nacional, Prol. de Carpio y Plan de Ayala S/N Col. Santo Tomás Alc. Miguel Hidalgo, Ciudad de México 11340, Mexico; alvarohr280391@gmail.com (Á.O.H.-R.); luisgzepeda@gmail.com (L.G.Z.-V.); evargasvd@yahoo.com.mx (M.E.V.-D.); 3 Unidad Profesional Interdisciplinaria de Biotecnología, Instituto Politécnico Nacional, Av. Acueducto s/n Barrio La Laguna, Ticomán, Alc. Gustavo A. Madero, Ciudad de México 07340, Mexico; ygomezipn@hotmail.com

**Keywords:** hibiscus acid, *Salmonella*, traditional medicine, motility, flagella, antivirulence

## Abstract

Extracts of *Hibiscus sabdariffa* L. (commonly called Rosselle or “Jamaica flower” in Mexico) have been shown to have antibiotic and antivirulence properties in several bacteria. Here, an organic extract of *H. sabdariffa* L. is shown to inhibit motility in *Salmonella enterica* serovars Typhi and Typhimurium. The compound responsible for this effect was purified and found to be the hibiscus acid. When tested, this compound also inhibited motility and reduced the secretion of both flagellin and type III secretion effectors. Purified hibiscus acid was not toxic in tissue-cultured eukaryotic cells, and it was able to reduce the invasion of *Salmonella* Typhimurium in epithelial cells. Initial steps to understand its mode of action showed it might affect membrane proton balance.

## 1. Introduction

The spread of multi-drug-resistant (MDR) bacteria is a global public health problem because the treatment of infections caused by these strains has been complicated in recent years, which has led to an increase in the number of deaths and prolonged stays of patients within hospitals [1,2]. In a catastrophic scenario, it is estimated that by the year 2050, approximately 10 million people will die each year from MDR bacterial infections. It is estimated that this number will be higher than cancer-related deaths (approximately 8.2 million deaths) [3,4,5]. This represents a great challenge for the scientific community, specifically in the design and discovery of new efficient and specific drugs [2,6].

In the search for new drugs for the treatment of diseases caused by MDR bacteria, novel strategies have been proposed such as antivirulence therapy [7,8]. This is based on inhibiting virulence mechanisms without intervening in the main metabolic pathways that are essential for bacterial viability. To understand how these drugs work, one must consider that virulence factors are elements that allow bacteria to infect and colonize a particular host; these factors are expressed under specific environmental conditions that are necessary for the pathogenesis process [7,9]. Virulence factors may be associated with the membrane, cytoplasm, or secreted once the pathogen has been established in the host cell. These allow motility, adhesion, evasion of the host immune system; adaptation to the environment through metabolic, physiological, or morphological changes; as well as inducing cell death of host cells [10]. Considering the high number of virulence factors that can be blocked to decrease bacterial pathogenicity, it is believed that antivirulence therapy has the potential to control and treat infections caused by pathogenic bacteria [7,11].

*Salmonella enterica* is a bacterium that is commonly acquired by the consumption of contaminated food and water; most of the serovars cause diarrhea in humans and also infects domestic animals, while a few serovars cause typhoid or paratyphoid fever in humans [12]. Some of the virulence factors in this pathogen include two type III secretion systems (T3SS) that favor internalization and colonization in the host [13]. Motility is also intimately related to *Salmonella* virulence, as it is required for the bacteria to successfully reach the small intestine and penetrate the intestinal mucosa [14,15]. Once the pathogen comes into contact with either M cells or enterocytes, the T3SS-1 encoded in the *Salmonella* pathogenicity island-1 (SPI-1) is expressed, injects effector proteins into the host cells, and modifies the cytoskeleton, favoring the internalization of the bacteria [13,16]. A possibility to avoid infection by *Salmonella* would be to block these virulence factors (motility and/or T3SS-1). Hypothetically, this would modulate the bacterial pathogenesis, making these bacteria more vulnerable to the host immune system [7,11,17].

In recent years, the potentiality of plant metabolites has been studied to mitigate bacterial virulence [11]. Some of these plants are also used for cooking or as herbal infusions, while others are used in the industry, and many have been tested for antimicrobial capabilities. The Roselle, commonly known as hibiscus or “Jamaica flower” (*Hibiscus sabdariffa* L.), has been used as an additive for food and beverages and in traditional medicine for the treatment of some illnesses [18,19,20]. In addition, it has been attributed pharmacological properties [18,19]. Among the most studied components in this plant are organic acids, anthocyanins, and flavonoids. To expand the study of Roselle, in this work, the antivirulence capacity against *Salmonella enterica* of subinhibitory concentrations of an organic extract of *H. sabdariffa* L. calyxes was tested. The results showed that this extract inhibited the motility of two *Salmonella* serovars, and by using chemical methods, the compound responsible for this inhibition was found to be the hibiscus acid (HA).

## 2. Results

### 2.1. Antimicrobial Activity of H. sabdariffa L. Extracts

**Figure 1:**
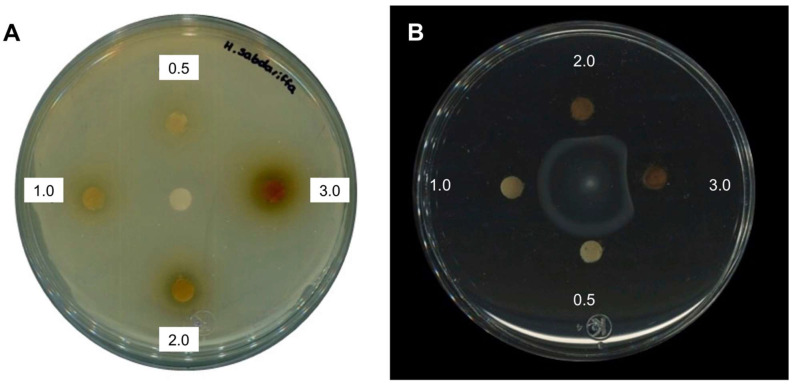
Antimicrobial and inhibitory activities of *H. sabdariffa* L. organic extract on *S.* Typhimurium. (**A**) Antimicrobial capacity and (**B**) inhibition of bacterial motility of *H. sabdariffa* L. extract at 0.5, 1, 2, and 3 mg/disk.

**Figure 2:**
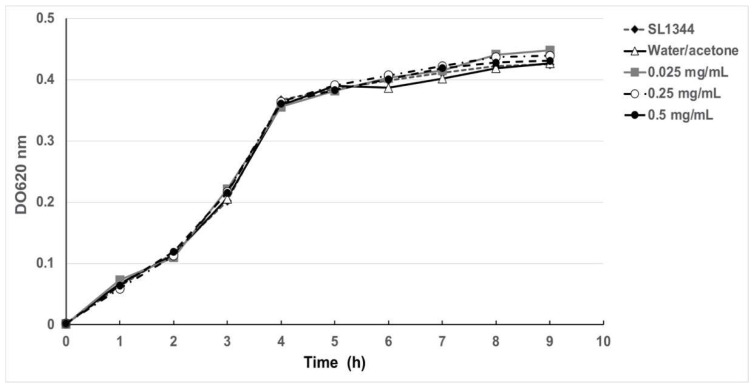
Effect of the organic *H. sabdariffa* L. extract on the growth of *S.* Typhimurium SL1344. Extracts at the indicated concentrations were incubated at 37 °C in agitation (200 rpm) and the optical densities at 620 nm were obtained for each one every hour for 10 h on a plate reader in a 200 mL volume per well. Control included the addition of water:acetone (50/50) to the same volume as that used with the extract.

A previous study described that an organic extract of *H. sabdariffa* L. had antimicrobial activity for several bacteria species including *Salmonella* [21,22,23]. To corroborate these results with the extracts obtained in this study, a similar experiment was performed showing that concentrations of 2 and 3 mg/mL indeed inhibited bacterial growth in Muller–Hinton agar. However, in order to test whether these extracts have any antivirulence properties, lower concentrations were tested on plate growth and in a growth curve of *Salmonella enterica* ser. Typhimurium SL1344. As shown in Figure 1A and Figure 2, extracts at concentrations of 0.025, 0.25, 0.5, and 1 mg/mL did not inhibit bacterial growth, while using 2 and 3 mg/mL showed a slight reduction in growth as expected. Taken together, the results showed that subinhibitory concentrations of *H. sabdariffa* L. organic extracts do not affect *S.* Typhimurium growth.

### 2.2. Anti-Motility Activity of H. sabdariffa L. Calyxes

**Figure 3:**
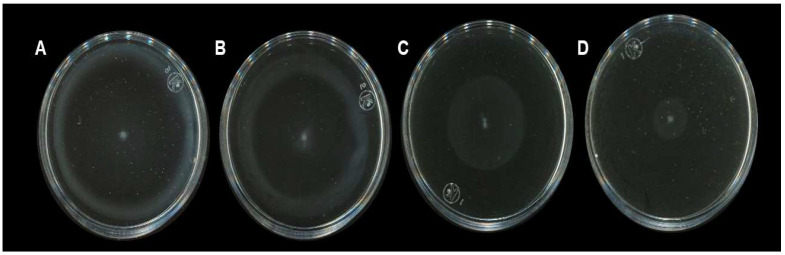
Effect of the organic *H. sabdariffa* L. extract on the motility of *S.* Typhi 6539. Motility plates were prepared with *H. sabdariffa* L. extracts at the following concentrations: (**A**) 0, (**B**) 0.125, (**C**) 0.25, and (**D**) 0.50 mg/mL. Plates were inoculated with 5 μL of bacterial culture in the center of the plates and incubated at 37 °C for 6 h. Plates were scanned over a black background to highlight the bacterial growth. Panel A shows the solvent control (water:acetone, 50/50).

To test if the *H. sabdariffa* L. extracts inhibited bacterial motility, these were used in a motility model with *S.* Typhimurium. The results shown in Figure 1B suggest that the motility was reduced even at low extract concentrations. To determine whether this effect on motility could also be observed with another *Salmonella* serovar, *Salmonella enterica* ser. Typhi ATCC 6539 was used. The results in Figure 3 show that the bacterial motility was also inhibited in *S.* Typhi in a concentration-dependent manner with *H. sabdariffa* L. extracts. A similar result was observed with *H. sabdariffa* L. specimens from two different sources.

### 2.3. Isolation of Secondary Metabolites from H. sabdariffa L.

To isolate the metabolite responsible for inhibiting *Salmonella* motility, the *H. sabdariffa* L. crude extract was analyzed by thin-layer chromatography (TLC) using methanol (MeOH): chloroform (CH_2_Cl_2_): ethyl acetate (EtOAc) (4:3:3) as the eluent and was also monitored by UV–vis at 254 nm and nuclear magnetic resonance (NMR). Consecutively, 7 g of the crude extract was subjected to column chromatography (CC) using a gradient system of hexane:EtOAc:MeOH in different ratios (described in Supplementary Table S1). Each obtained fraction was tested for biological activity (i.e., as a motility inhibitor), pooled, and called “fraction A”, then subjected to an additional fractionation (information about this procedure is provided in Supplementary Table S2). Similarly, fractions with biological activity were pooled together and called “fraction B” and subjected to a final round of fractionation (Supplementary Table S3). Pooled fractions with biological activity were called “fraction C” and analyzed as indicated in the following sections.

#### Characterization of HA by NMR and Mass Spectrometry

**Figure 4:**
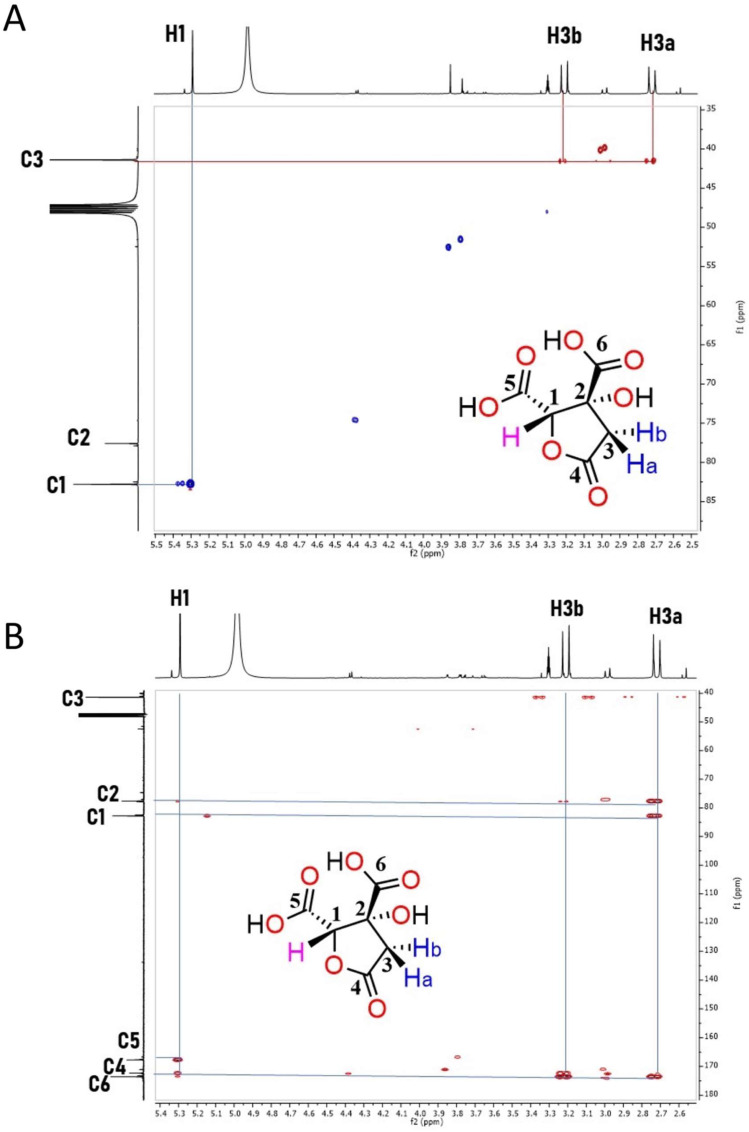
NMR analyses. (**A**) Homonuclear single quantum correlation (gHSQC) spectrum of hibiscus acid showing one-bond H-C correlations. (**B**) Heteronuclear multiple bond correlation (HMBC) spectrum showing heteronuclear long-range, 2- and 3-bond correlations. In both panels, the chemical formula is shown as an inset.

Pooled fractions were analyzed by NMR as described in the Methods section. The results in Figure 4 show that by using NMR and mass spectrometry, the main compound in the active fraction that retained an anti-motility activity for *S.* Typhi and *S*. Typhimurium is HA (see also Supplementary Figure S1). In the ^1^H NMR spectrum a doublet signal (^1^H, ^2^J = 17.2 Hz) appears at δ 2.72 corresponding to the diastereotopic proton H-3a, and at δ 3.21, its geminal proton H-3b can be observed as a doublet (^1^H, ^2^J = 17.2 Hz). At δ 5.30, a singlet signal (^1^H) appears corresponding to H-1. In the proton-decoupled ^13^C NMR spectrum, the expected six signals of HA are observed. At δ 175.09 and δ 169.23, signals for carboxylic acids C6 and C5, respectively, are shown. The signal at δ 173.82 corresponds to the carbonyl group (C4) of the lactone. At δ 84.32 and δ 79.11 ppm, signals for C1 and C2, respectively, are observed, while the signal at δ 42.90 belongs to the methylene C3. The ^1^H and 13C NMR spectra were correlated and assigned by two-dimensional experiments (gHSQC and gHMBC). Mass spectrometry analysis afforded *m*/*z* 189.89, which corresponds to ion [M-1]^+^; additionally, *m*/*z* 379.01 for the HA dimer was observed. Taken together, this structural analysis shows that HA is present in the collected fractions that are able to inhibit *Salmonella* motility.

### 2.4. Antimicrobial and Anti-Motility Activities of HA

**Figure 5:**
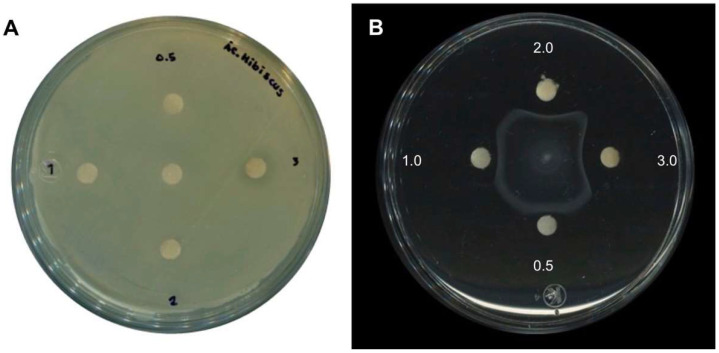
Antimicrobial and inhibitory activities of hibiscus acid on *S.* Typhimurium. (**A**) Antimicrobial capacity and (**B**) inhibition of motility of purified hibiscus acid at 0.5, 1, 2, and 3 mg/disk, respectively. 4% DMSO was used as solvent control in panel A, and it is shown in the center of the plate.

In order to corroborate that HA has antimicrobial and anti-motility activities, the purified compound was tested as described above for the *H. sabdariffa* L. extract. The results in Figure 5 show that the purified HA inhibited growth when high concentrations (3 mg/disk) were tested, and when lower concentrations (0.5 mg/disk) were tested, the inhibition of mobility was observed. Taken together, these results show that HA is responsible for inhibiting both bacterial growth and motility.

### 2.5. HA Partially Affects Flagellin Secretion

**Figure 6:**
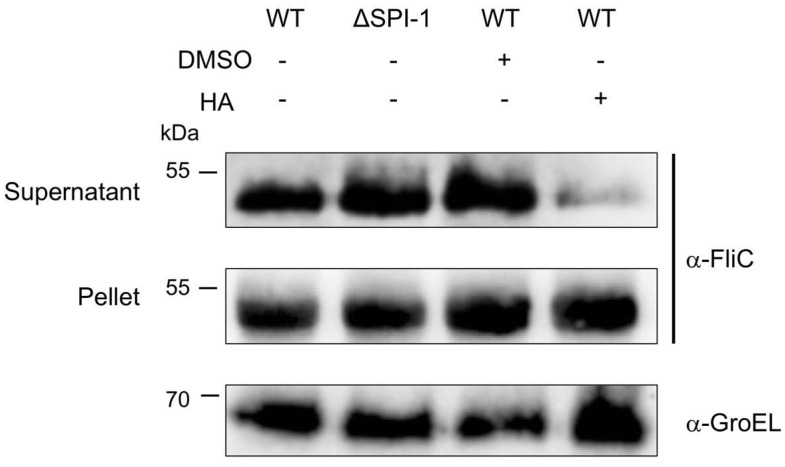
Hibiscus acid reduces flagellin secretion. Secreted proteins were obtained from the indicated cultures with the addition or not of HA, and flagellin was detected by Western blots with anti-FliC antibodies. Intracellular FliC and GroEL proteins were also detected. Proteins detected in the supernatant and the pellet are shown. DMSO shows the solvent control and HA the addition of hibiscus acid (1 mg/mL).

To understand how HA inhibits bacterial motility biogenesis and secretion of the flagellin, FliC in *S.* Typhimurium SL1344 was detected by Western blot. The results showed that the addition to HA to the media inhibited FliC secretion but not its synthesis (Figure 6). Taken together, these results suggest that this compound might be acting to inhibit a component of the flagella type-III secretion system (fT3SS) involved in the secretion of FliC.

### 2.6. HA Partially Inhibits Secretion of T3SS Effector Proteins

**Figure 7:**
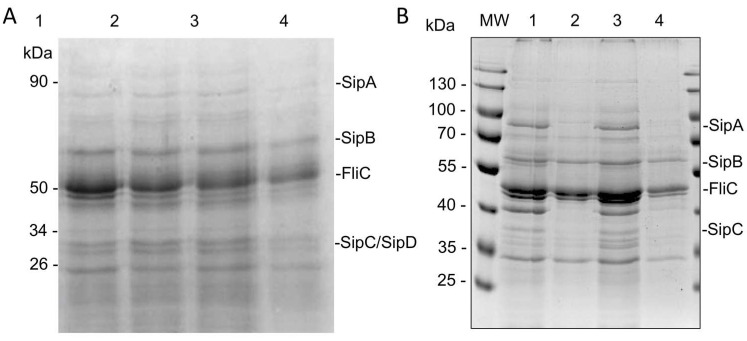
Hibiscus acid reduces T3SS-1 effector secretion. Secreted proteins were obtained from the indicated cultures in SPI-1-inducing conditions with the addition or not of *H. sabdariffa* L. extract (**A**) and hibiscus acid (**B**). (**A**) Coomassie-stained gel of secreted proteins from *S.* Typhimurium SL1344 in SPI-1-inducing conditions, lanes: 1, no DMSO added; 2, solvent control water/acetone (50:50); 3, *H. sabdariffa* L. extract (0.25 mg/mL); 4, *H. sabdariffa* L. extract (0.5 mg/mL). (**B**) Coomassie-stained gel of secreted proteins from *S.* Typhimurium SL1344 in SPI-1-inducing conditions, lanes: MW, molecular weight markers; 1, wild-type strain without DMSO added; 2, ΔSPI-1 strain; 3, wild-type strain with solvent control (4% DMSO); 4, wild-type strain with purified hibiscus acid (1 mg/mL).

Given the previous results on the effect of the HA on the flagella biogenesis, its effect was tested in the secretion of effector proteins encoded in the SPI-1. As shown in Figure 7A, the secretion of *S.* Typhimurium effectors was reduced but not eliminated when *H. sabdariffa* L. extracts were used. To corroborate this observation, the secretion of proteins was tested with purified HA, and, as seen Figure 7B, a marked reduction in secreted proteins was observed. These results suggest that both the *H. sabdariffa* L. extract and the HA affect the secretion of proteins in *Salmonella*.

### 2.7. The Hibiscus Acid Is Not Cytotoxic for Human Epithelial Cells

**Figure 8:**
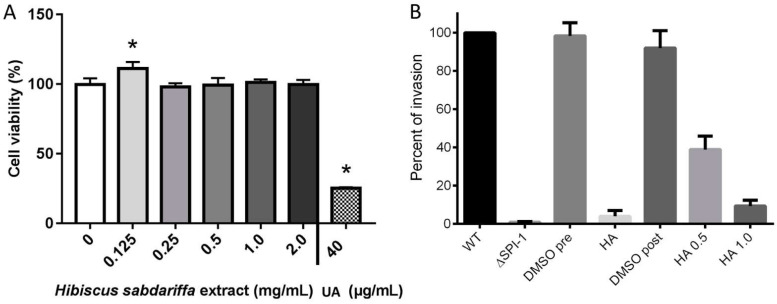
The hibiscus acid is not toxic for cultured epithelial cells and reduces invasion of *S.* Typhimurium. (**A**) Cell monolayers were treated with the indicated concentrations of hibiscus acid, and the viability of the cells was assessed with MTT. The results are presented as means and standard deviations of three replicates. (*) (**A**) *p* < 0.05 was considered as statistically significant between treated and non-treated cells, and it was calculated by one-way ANOVA with Dunnett’s post-test. UA, 40 µg/mL ursolic acid used as death control. (**B**) Purified HA dissolved in 4% DMSO was added during the induction of SPI-1 conditions (pre) or during the invasion assay (post) in HeLa cells in two concentrations, 0.5 mg/mL and 1 mg/mL (HA 0.5 and HA 1.0, respectively). Added volumes of HA were 7.5 to 15 µL per well from a 75 mg/mL stock solution to the indicated concentrations. Numbers of invading bacteria were obtained from four different experiments performed in duplicates, and the results are presented as percentage of invasion compared with the wild-type strain obtained in SPI-1-inducing conditions. Wild-type (WT) invasion was considered as 100%, and the rest of the conditions were compared with this for each assay, and then the percentages of each repeat were averaged. DMSO was added either during the inducing conditions (DMSO pre) or during the invasion assay (DMSO post).

To investigate whether the purified HA is cytotoxic on HeLa cells, cytotoxicity assays were conducted. The results showed that the tested concentrations did not have a negative effect on the viability of the cells. The less concentrated solution assessed (0.125 mg/mL) had a slight significative effect on the cultured cells, increasing their proliferation (Figure 8A). Ursolic acid was used as a positive control for cellular death. These results showed that HA has no cytotoxic activity at the tested concentrations.

### 2.8. HA Reduces Invasion of Salmonella

To determine whether the purified HA affects epithelial cell invasion by *S.* Typhimurium, two protocols were followed: (1) by adding this compound during the SPI-1-inducing conditions growth in LB-Miller and (2) by adding it to the cultured cells prior to the addition of SPI-1-induced *Salmonella* for the invasion assay. The results shown in Figure 8B demonstrated that both the addition of HA before and during the infection of HeLa cells strongly reduced *Salmonella* invasion. Taken together these results demonstrate that the HA has antivirulence properties by reducing the invasion of this bacterium to epithelial cells. 

### 2.9. HA Induces Metabolic Alterations on Salmonella

To investigate the effect of HA on the metabolism of *Salmonella*, the changes in extracellular pH after the addition of this compound to the media were monitored. Changes in the pH are closely related to the bacterial cell metabolic status [24,25]. 2,4-Dinitrophenol (DNP) is a well-known protonophore that transports protons across biological membranes, dissipating the electrochemical protons gradient [26,27]. The addition of 4% DMSO did not change the pH of either the medium or the cellular suspension, while the addition of HA or DNP caused a slight acidification (pH ~5.5 to 6.8). The pH was measured for 60 min, and the results showed that both HA and DNP provoked proton consumption, resulting in an increase in extracellular pH (Supplementary Figure S2). Surprisingly, the extracellular pH increase induced by HA was significantly higher than that observed with DNP at sublethal concentrations. Taken together, these results suggest that the HA alters the proton-transport processes at the membrane level in Salmonella.

To determine whether the change in pH has an effect on bacterial motility, the tests were repeated in soft LB agar prepared with the MOPS and HEPES regulators (1 mM, pH 7.0 in each case), and 0.5 mg/mL of *H. sabdariffa* L. organic extract was added. The results shown in the Supplementary Material (Supplementary Figure S3) demonstrate that even at a neutrally regulated pH of the media, the bacterial mobility was affected. These results suggest that mild acidic pH is not responsible for affecting the *Salmonella* motility.

## 3. Discussion

The antibacterial properties of the Roselle or hibiscus have been studied and shown to be effective against bacteria causing nosocomial infections such as methicillin-resistant *Staphylococcus aureus* (MRSA), *Staphylococcus epidermidis*, *Proteus vulgaris*, *Klebsiella pneumoniae*, *Pseudomonas aeruginosa*, and *Acinetobacter baumannii* [20,28,29]. Here, the antivirulence properties of *H. sabdariffa* L. organic extracts against *Salmonella enterica* were tested, and the compound responsible for this effect was isolated.

As mentioned above, *H. sabdariffa* L. extracts have antimicrobial activity; therefore, lower concentrations were tested for their ability to inhibit bacterial motility. The initial test using lower than 1 mg/mL concentrations of the organic extract showed not to have an effect in bacterial growth of *S.* Typhimurium, but it affected this bacterium’s motility. Moreover, this effect was also extended to *S.* Typhi. Following a chemical workflow described in the Methods section, a major component in the fractions that retained the ability to inhibit *Salmonella* motility was detected, which showed to be the HA, as the obtained NMR data corresponded with those by Portillo-Torres and colleagues [22]. Despite the fact that many studies have been carried out with the extract (either organic or aqueous) of *H. sabdariffa* L. [18,19,20,28,29], only a few studies have been focused on HA [30,31,32,33,34]. In a few reports, it was shown to have inhibitory activity against α-amylase and α-glucosidase and also an anti-hypertensive effect in eukaryotes [30,32,33]. At first glance, these activities do not seem to have a relationship with either the biogenesis of either the fT3SS, the T3SS-1, or the motility, which might explain the effect shown by the results described here.

In regard to its antibiotic properties, Portillo-Torres and colleagues [23] showed that HA has activity against *Escherichia coli*, *S.* Typhimurium, *P. aeruginosa*, *S. aureus*, and *Vibrio cholerae*. In a more recent report, the use of small concentrations of HA showed to decrease the virulence of *P. aeruginosa* [34]. In this report, the authors proposed that the HA interacts with LasR inhibiting the quorum sensing (QS) in this bacterium. In order to explore the possible mechanism of action of the HA to inhibit *Salmonella* motility, the secretion of flagellin and alteration in metabolism were explored. In relation to the secretion of flagellin, the results showed that the FliC synthesis was not inhibited, suggesting that HA does not affect the regulatory network for the expression of this protein and perhaps that of the other components of flagellum. Thus, despite the fact that the flagella synthesis in *Salmonella* is regulated by QS [35] and that HA inhibits this system on other bacteria [34]. The results presented here suggest that the effect is on another level as the secretion of this protein was affected but not its synthesis, though this needs to be further studied. Our results suggest that HA might affect the secretion through the fT3SS secretory apparatus. Moreover, the secretion of SPI-1 T3SS effectors is also reduced and, similarly to flagellum, its synthesis is not affected when HA was added. This again implies that HA does not affect the expression of the SPI-1 effectors but their secretion.

Both pieces of data suggest that HA might act by reducing the secretory process of both flagellin and SPI-1 effectors. The addition of HA either during bacterial growth or just prior to the invasion assay dramatically reduced the invasion of *Salmonella*. As expected for the initial results mentioned above, the addition of HA during the incubation period did not affect the bacterial growth, supporting our previous data that this amount does not have an effect during the growth curve. In fact, adding the HA during the invasion assay suggests that this compound could either block the secretory channel or inhibit the activity of the ATPase at the base of the T3SS [36]. Thus, some of the questions that arise are: does HA act on the surface of the bacterium, or is the HA able to penetrate and gain access to both the periplasm and the cytoplasm of the bacterium?

As an initial step to understanding how HA might affect the secretion of both flagellin and SPI-1 effectors, the changes in extracellular pH were measured and compared with those induced by DNP, a well-known protonophore. pH reports the chemical activity of protons, relevant in metabolic reactions (redox), mineral dissolution, etc.; changes in it also reflect the activity of enzymes and other cellular processes such as motility [37]. Both, HA and DNP showed a similar effect by lowering the extracellular pH immediately after these were added to the bacterial suspension. DNP has been shown to dissipate the proton electrochemical protons gradient in mitochondria, resulting in proton motive force inhibition in bacteria [38]. The results showed that, after a few minutes, the extracellular pH started to increase in both cases. This may be the result of the entry of protons that migrate from a higher concentration area (the extracellular environment) to a lower concentration area (the periplasm), perhaps through porins located in the bacterium’s outer membrane. Of note, the increase was higher with HA than with DNP. This difference in the consumption of protons between the treatment with HA and DNP might be due to their chemical nature. Previous studies have demonstrated that a change in pH across the cell membrane of only 0.2 units is sufficient to achieve variations in proton motive force [39]. The HA may affect the proton motive force generation, which is closely related to flagellum functionality, which could explain the observed effects in mobility. An alternative is that the acidic pH causes the flagella to be shed from the bacterial surface as has been described [40]. As described by the authors, the latter is achieved at very low pH (pH = 2.0), which is not reached by the addition of either DNP or HA in the experiments described here; thus, this might not be the case. On the other hand, changes in pH have an effect in both the expression of flagellar genes and motility in enterobacteria [41]. In this way, *E. coli* motility is sustained in a pH range of 6.0 to 7.5, while *Salmonella* motility is slightly better in a pH range of 6.0 to 6.5. The results presented here show that the addition of HA mildly acidifies the media (~pH 5.5 to 6.8); therefore, this change might not be responsible for affecting the motility. Moreover, the addition of *H. sabdariffa* L. organic extract affects *Salmonella* motility even in the presence of pH regulators (such as HEPES or MOPS), which reinforces the idea that mild acidic pH is not responsible of affecting the motility. Although this test has not been performed with HA, the results presented demonstrate that it is the inhibitory compound for this bacterium motility and it is possible that the salt of HA formed in solution is affecting the consumption of protons, thus disturbing the flagella motion. In the case of the T3SS-1, the secretion of the effectors might also be affected by the change in this proton balance with an effect in the dedicated ATPase involved in their secretion, which in turn would impact the invasion process. However, more studies are needed to confirm these hypotheses.

Additionally, results presented here showed that HA is not cytotoxic for cultured epithelial cells suggesting that it is safe for humans or animals, although low concentrations of HA seem to increase cell proliferation. This latter could be due to previously described antioxidant and antiapoptotic effects of *H. sabdariffa* L. extracts [42]. However, this has not been shown for HA. Moving to the use of pure HA in animals or humans will require more studies, not only in cultured cells but in animal models.

## 4. Materials and Methods

### 4.1. H. sabdariffa L. Extract

The experimental research with the cultivated plant complies with institutional, national, and international guidelines and legislation. *H. sabdariffa* L. is easy to acquire in any public market in Mexico, and it is usually sold dried. Additionally, it is also produced in many parts of this and other Latin-American countries. Specimens of this plant are found in the collection “ENCB Herbario Jerzy Rzedowski y Graciela Calderón“ at the herbarium of our institution with the number BC007 (https://www.encb.ipn.mx/herbario/ (accessed on 11 October 2021)), and locations where it is produced in Mexico have been documented by the CONABIO (https://enciclovida.mx/especies/165257-hibiscus-sabdariffa (accessed on 11 October 2021)). The calyxes of *H. sabdariffa* L. used in this work were obtained from two sources: one batch was from a commercial source sold under the brand “Comercial Mexicana” in a convenience store, and the other batch was originally from Cuba and was obtained in a public market located behind the Metropolitan Cathedral in Mexico City. Fifty grams of calyxes previously dehydrated were macerated at room temperature with 500 mL of acetone (Sigma-Aldrich, St. Louis, MO, USA) for 24 h. Subsequently, the organic extract obtained was filtered and the solvent was evaporated to obtain 7 g of dried extract (total yield of 14%).

### 4.2. Isolation of Secondary Metabolites from H. sabdariffa L.

The *H. sabdariffa* L. crude extract was analyzed by TLC using MeOH: CH_2_Cl_2_: EtOAc (4:3:3) as the eluent and was also monitored by UV–vis 254 nm and ^1^H NMR. Consecutively, 7 g of the crude extract was subjected to column chromatography (CC) using a gradient system of hexane:EtOAc:MeOH in different ratios as described in Supplementary Table S1. Each fraction obtained was tested for biological activity and analyzed spectroscopically. Other fractionations used are described in Supplementary Tables S2 and S3.

### 4.3. NMR and Low-Resolution Mass Spectrometry

NMR characterization was performed at 600 MHz for proton (^1^H) and 150 MHz for carbon (^13^C) on a Bruker 600 AVANCE III HD spectrometer (Bruker Ltd., Billerica, MA, USA) equipped with a 5 mm 1H / D TXI probe-head with *z*-gradient. Chemical shifts are indicated from the signal of TMS used as reference (0 ppm); methanol-D_4_ was used as a solvent to prepare the samples. The low-resolution mass spectra (LRMS) were recorded on the Bruker amaZon speed spectrometer (Bruker Ltd., Billerica, MA, USA) with an ion trap using the ESI ionization method. 

### 4.4. Bacterial Strains and Growth Conditions

*Salmonella* Typhi ATCC 6539 was kindly supplied by Dr. Guadalupe Aguilera-Arreola (Departamento de Microbiología, ENCB-IPN), and it was used for growth and motility assays. *S*. Typhimurium SL1344 wild-type and its derivative ΔSPI-1 were provided by Dr. Víctor H. Bustamante Santillán (Instituto de Biotecnología, UNAM). These strains were used for secretion and invasion assays (see below). Bacterial strains were streaked on LB-Miller agar and incubated from 18 to 24 h at 37 °C. Subsequently, a colony of each culture was taken and inoculated in LB culture medium, which was incubated at 37 °C in agitation. 

### 4.5. Antimicrobial Activity and Growth Curves

The antimicrobial capacity of the total organic extract of *H. sabdariffa* L. was determined by performing growth curves with the strains of *S*. Typhimurium SL1344 and ∆SPI-1 in 96-well plates (Corning, Corning, New York, NY, USA) containing 180 μL of LB inoculated with 10 μL of each of the cultures previously adjusted to 1 × 10^8^ CFU/mL. Additionally, 10 μL of *H. sabdariffa* L. extract in increasing concentrations (0.025, 0.25, and 0.5 mg/mL) were added to the wells in the microplate. Solvent control included water/acetone (50:50) and added to the plate in a volume similar to that added for the highest concentration. The 96-well plate was incubated at 37 °C in agitation (225 rpm), and absorbance readings were obtained every hour for 10 h on a plate spectrophotometer (Multiskan Go, Thermo Scientific, Waltham, MA, USA) at 600 nm. Please note that the aeration is lower than that obtained in a flask. The analysis of the growth curve was performed with the GraphPad Prism 5 program.

To determine the antimicrobial activity on solid media, a disk diffusion test was performed as described by the CLSI procedures [43]. Either the strain *S.* Typhi ATCC 6539 or *S*. Typhimurium SL1344 were adjusted to 1 × 10^8^ CFU/mL and 100 μL were inoculated in Muller–Hinton agar plates; sterile filter paper discs (Whatman no. 1, Whatman, Maidstone, UK) were prepared by placing the organic extracts or the purified HA in the following concentrations: 0.5, 1, 2, and 3 mg/disk. Plates were incubated at 37 °C for 18 h and the inhibition halos were measured.

### 4.6. Motility Assay

Motility assays were performed on semi-solid LB agar (agar 0.2%) following a previously described protocol [14]. The *H. sabdariffa* L. extract was tested in concentrations 0.125, 0.25, 0.5, 1, 2, and 3 mg/mL in 100 µL of water/acetone (50:50) as solvent. Five to ten microliters of an overnight culture of *S.* Typhimurium or *S.* Typhi were inoculated in the center of the plates and incubated at 37 °C. A 6 h monitoring was performed measuring the mobility diameter in mm. The plates were scanned for clearer images of the motility halos.

### 4.7. Secretion Assay

Secretion of type III secretion effectors was performed as previously described [14,44]. Briefly, 50 μL of an overnight culture was inoculated in multiple tubes with 5 mL of LB-Miller, one of the tubes was used as a control, and 0.5 mg/mL of total extract of *H. sabdariffa* L. was added to the others or HA dissolved in 50 μL of water/acetone (50:50). The cultures were incubated for 9 h at 37 °C in agitation (225 rpm) and the optical density was measured at 600 nm. The culture was centrifugated for 20 min at 13,000 rpm at 4 °C, the supernatant was transferred to a 15 mL tube and approximately 800 μL of trichloroacetic acid (TCA) was added to a final concentration of 10%, homogenized, and stored all night at 4 °C to favor the precipitation of the proteins. The supernatants were centrifugated at 13,000× *g* for 30 min at 4 °C, and two washes were performed with 1 mL of cold acetone, decanting the supernatant. Once the pellets were air-dried, 50 μL of Laemmli buffer and 5 to 10 μL of NaOH 1 M were added to resuspend the pellet and neutralize the pH, respectively. On the other hand, 100 μL of Laemmli buffer was added to the pellets and both samples were heated at 95 °C for 5–10 min. Samples were loaded onto a 12% SDS-PAGE and either stained with Coomassie stain or transferred to a nitrocellulose membrane, and tested in a Western blot with anti-GroEL (1:5000) (Abcam, Cambridge, UK) or anti-FliC (see below). Subsequently, 3 washes with 25 mL of Tris-borate-saline-Tween buffer (TBS-T) were made to each membrane and blocked with secondary antibodies anti-IgG-HRP (1:4000) or protein G-HRP (1:5000) (Invitrogen, Waltham, MA, USA). The proteins were detected by immunoblot analysis using the kit Novex^®^ ECL Chemiluminiscent Substrate Kit (ThermoFisher, Waltham, MA, USA) and observed in a Chemidoc MP Imaging system (BioRad, Hercules, CA, USA).

### 4.8. Cytotoxicity Assays

To evaluate the cytotoxicity of the purified HA, the HeLa cell line from the American Type Culture Collection (ATCC) was used. Cells were maintained in Dulbecco’s modified Eagle’s medium (DMEM, Sigma-Aldrich, St. Louis, MO, USA) supplemented with 10% fetal bovine serum at 37 °C in a humidified 5% CO_2_ atmosphere. The cytotoxicity was assessed by using 3-(4,5-dimethylthiazol-2-yl)-2,5-diphenyltetrazolium bromide (MTT, Sigma-Aldrich, St. Louis, MO, USA) assay as described previously [45]. Briefly, confluent monolayers of HeLa cells prepared in a 24-well plate were exposed to 0.125, 0.25, 0.5, 1, and 2 mg/mL HA and incubated for 24 h at 37 °C and 5% CO_2_. Then, 100 µL of a 1 mg/mL MTT solution was added and incubated for another 2 h. Afterward, the formed formazan precipitates were solubilized with 200 µL of DMSO. Absorbance was quantified at 570 nm in a SpectraMax M3 microplate reader (Molecular Devices, San Jose, CA, USA). Monolayers without extract were taken as viability controls and monolayers treated with 40 µg/mL ursolic acid were used as a positive control of cellular death.

### 4.9. Invasion Assays

*Salmonella* invasion assays were performed in cultured HeLa epithelial cells with the SL1344 strain and an ΔSPI-1 as described previously with slight modifications [14]. Briefly, exposure to purified HA was performed with two different methods: (a) by adding it to a final concentration of 1 mg/mL (*w*/*v*) to 10 mL of LB-Miller during the SPI-1-inducing conditions (3.5 h, 225 rpm at 37 °C) or (b) by adding it to the final concentrations of 0.5 or 1 mg/mL (*w*/*v*) in a 24-well plate with HeLa cells prior to the addition of bacteria. The numbers of invading bacteria were obtained from four different experiments performed in duplicates, and the results are presented as a percentage of invasion compared with the wild-type strain obtained in SPI-1-inducing conditions. Thus, invasion by the wild-type strain was considered as 100%; the rest of the conditions were compared with this for each assay, and then the percentages of each repeat were averaged. 

### 4.10. Western Blot for Flagellin

In order to narrow the effect of the hibiscus acid on the flagellum, a Western blot to detect flagellin FliC was performed in bacterial growth in SPI-1-inducing conditions as described before [14]. Briefly, once the bacteria reached the inducing conditions, all the culture was centrifugated (15,000× *g* for 20 min at 4 °C) and the supernatant and pellet were separated. Pellets were resuspended in Laemmli buffer. Proteins were precipitated as described above. Proteins samples (5 μL of each sample) were separated into 10 or 12% SDS-PAGE gels and transferred to a PVDF membrane (Millipore, Burlington, MA, USA). Membranes were blocked with 3% (*w*/*v*) skim-milk solution for 2 h, then 3 washes were carried out with 25 mL of TBST. Subsequently, the membranes were incubated either with 10 mL of a solution of anti-FliC (1:10,000) antibody (kindly provided by Dr. Francisco Javier de la Mora, Instituto de Fisiología Celular, UNAM) or with 10 mL of an anti-GroEL (Abcam, Cambridge, UK) antibody solution (1:10,000) for 1 h; then, 3 washes were performed with 25 mL of TBST and incubated with a solution of recombinant protein G-HRP (Invitrogen, Waltham, MA, USA) (1:5000) and finally washed 3 times with 25 mL of TBST. The membrane was developed using the Novex ECL Chemiluminescent Substrate Kit (Thermo Fisher, Waltham, MA, USA) and the images acquired in a Chemidoc Imaging System (Biorad, Hercules, CA, USA).

### 4.11. Metabolic Assay

A cellular suspension of *S.* Typhimurium was adjusted to 0.5 OD_600nm_ with LB medium. Then, 20 mL of the bacterial suspension was placed in 50 mL tubes and HA was added to a final concentration of 1 mg/mL (5 mM). Control conditions included DMSO and DNP (Sigma-Aldrich, St. Louis, MO, USA) at sub-lethal concentrations of 1.25 and 2.5 mM. All the tubes were incubated in agitation (120 rpm) at 25 °C and 2 mL samples were taken at 0, 10, 20, 30, and 60 min. The collected samples were centrifuged for 2 min at 10,000× *g* at room temperature, supernatant was moved to a new tube, and the pH was measured by using a conventional pH electrode (Hanna Instruments, Woonsocket, RI, USA). The assay was carried out twice with duplicates.

## 5. Conclusions

In summary, the results presented here show that HA isolated from *H. sabdariffa* L. is responsible for inhibiting *Salmonella* motility when used in concentrations lower than 1 mg/mL. This compound was also able to reduce *S.* Typhimurium invasion to cultured epithelial cells. Initial steps were taken to deduce how HA affects the physiology of *Salmonella*, but more experiments will be needed to accurately determine it. The results show that HA could be used as an antivirulence compound either as a food additive or as a complement for antibiotics treatment.

## Supplementary Materials

The following supporting information can be downloaded Figure S1: NMR and mass spectral data for purified hibiscus acid, Figure S2: Effect of HA and DNP on extracellular pH in *Salmonella* cultures, Figure S3: *Salmonella* motility in buffered media, Table S1: Composition of the mobile phase used for the fractionation of the total extract of *H. sabdariffa* L. by column chromatography and biological activity, Table S2: Composition of the mobile phase used for the fractionation of the “Fraction A” by column chromatography and biological activity, Table S3: Composition of the mobile phase used for the fractionation of “Fraction B” by column chromatography and biological activity.

## Sample Availability

Samples of the compound are available from the authors.
