# Supplementary material for: Hibiscus Acid from Hibiscus sabdariffa L. Inhibits Flagellar Motility and Cell Invasion in Salmonella enterica
Source: Molecules. 2022 Jan 20;27(3):655. doi: 10.3390/molecules27030655 (PMC8839027; doi:10.3390/molecules27030655)
Supplement: Supplementary file 1 [file molecules-27-00655-s001.zip › molecules-1525054-supplementary.pdf]

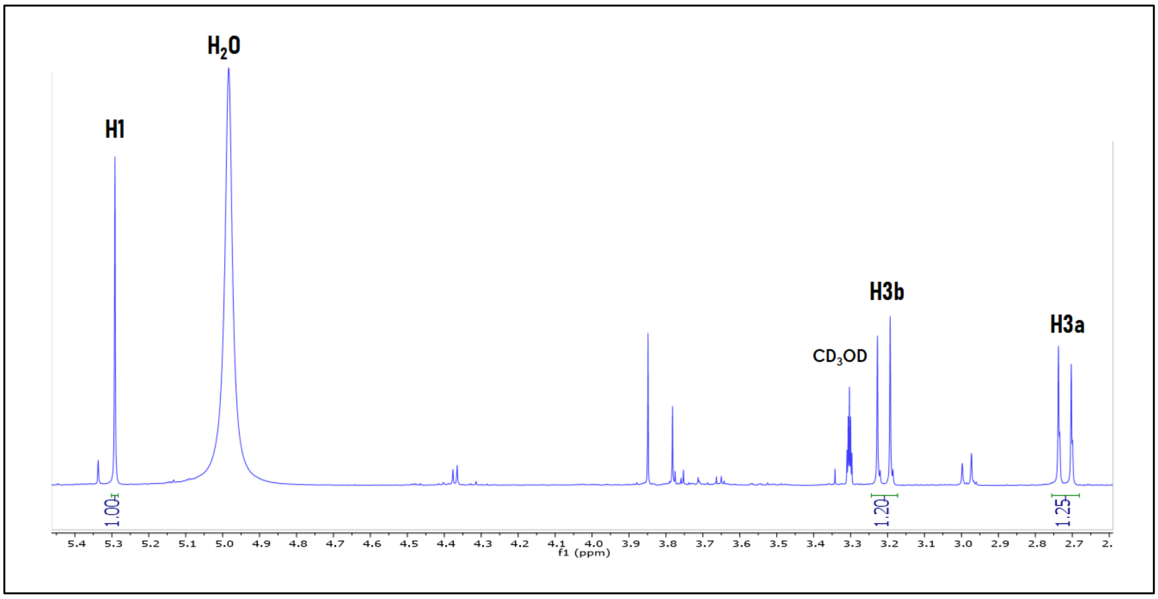
H1
H_2O
CD_3OD
H3b
H3a
1.00
1.20
1.25
f1 (ppm)

(a)

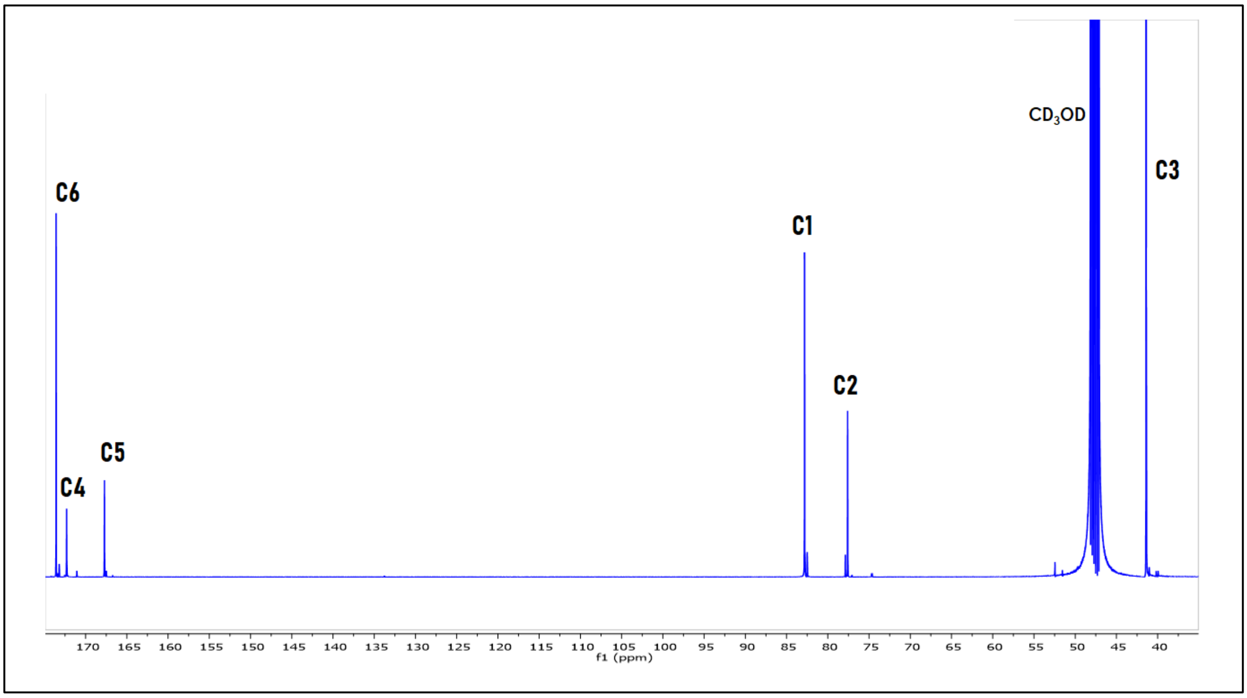
C6
C4
C5
C1
C2
CD_3OD
C3
f1 (ppm)

(b)

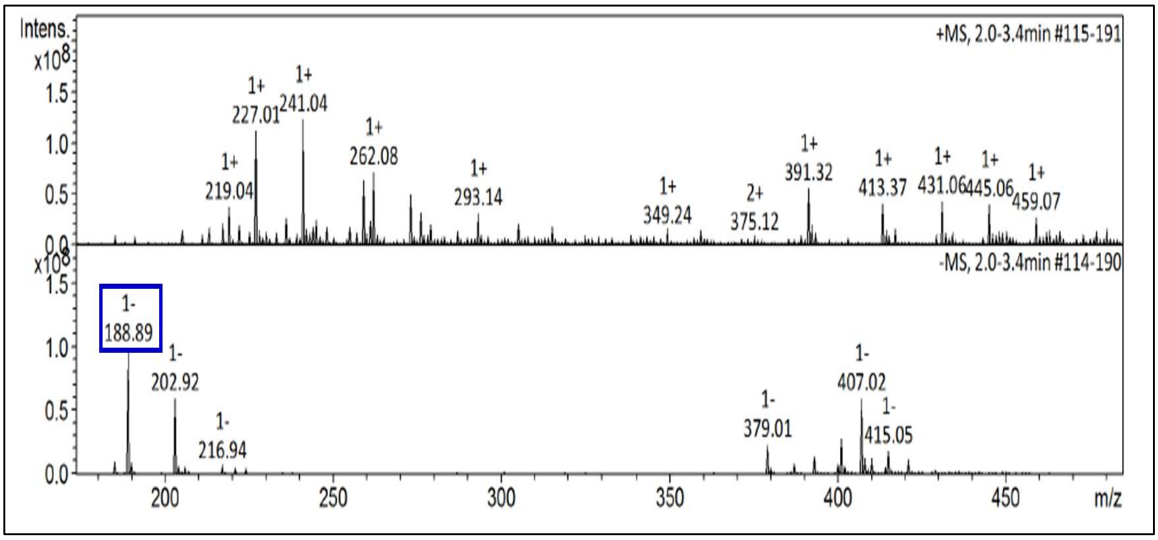

(c)

Supplementary Figure S1. NMR and mass spectrum data for purified hibiscus acid. (a) ^{1}H NMR spectrum (600 MHz, CD_3OD) of a sample of hibiscus acid. (b) ^{13}C NMR (150 MHz, CD_3OD) spectrum of hibiscus acid. (c) Positive (up) and negative (down) mass spectrum (ESI) of hibiscus acid.

Supplementary Figure 2

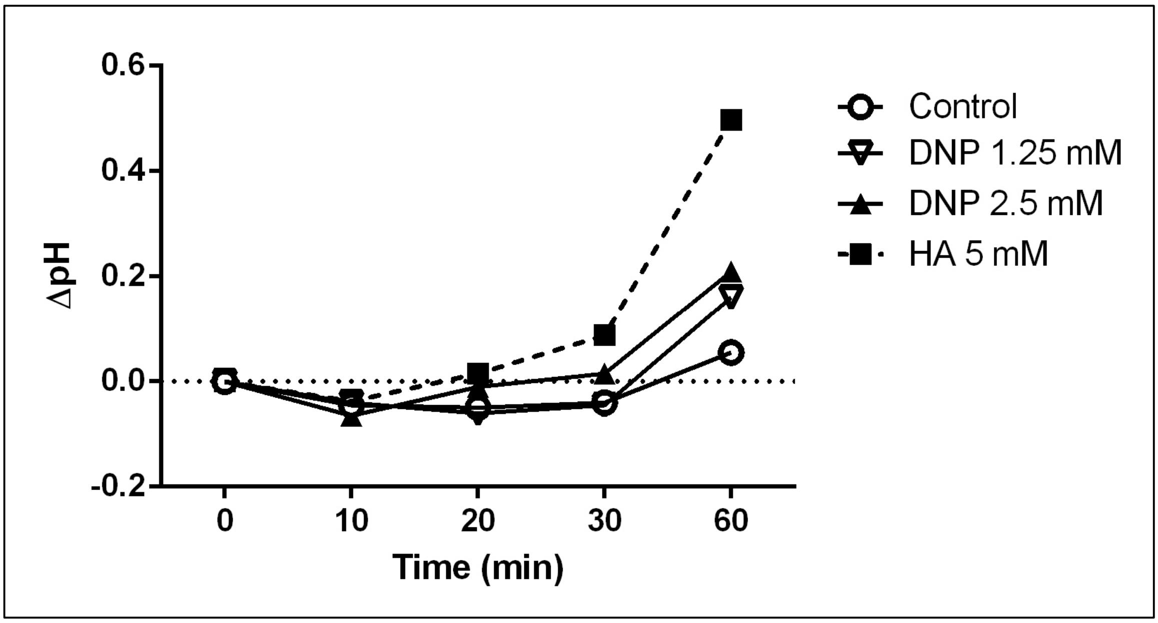

Supplementary Figure S2. Effect of HA and DNP on extracellular pH in *Salmonella* cultures. Represented is the difference in pH (ΔpH) between the starting point when the compounds were added (0 min) and the indicated time point for each case. Cultures were incubated at 25 °C in agitation (120 rpm) and pH was determined every 10 min as described in the Methods section. Results are the average of two independent experiments made in duplicates. 4% DMSO was used as a control.

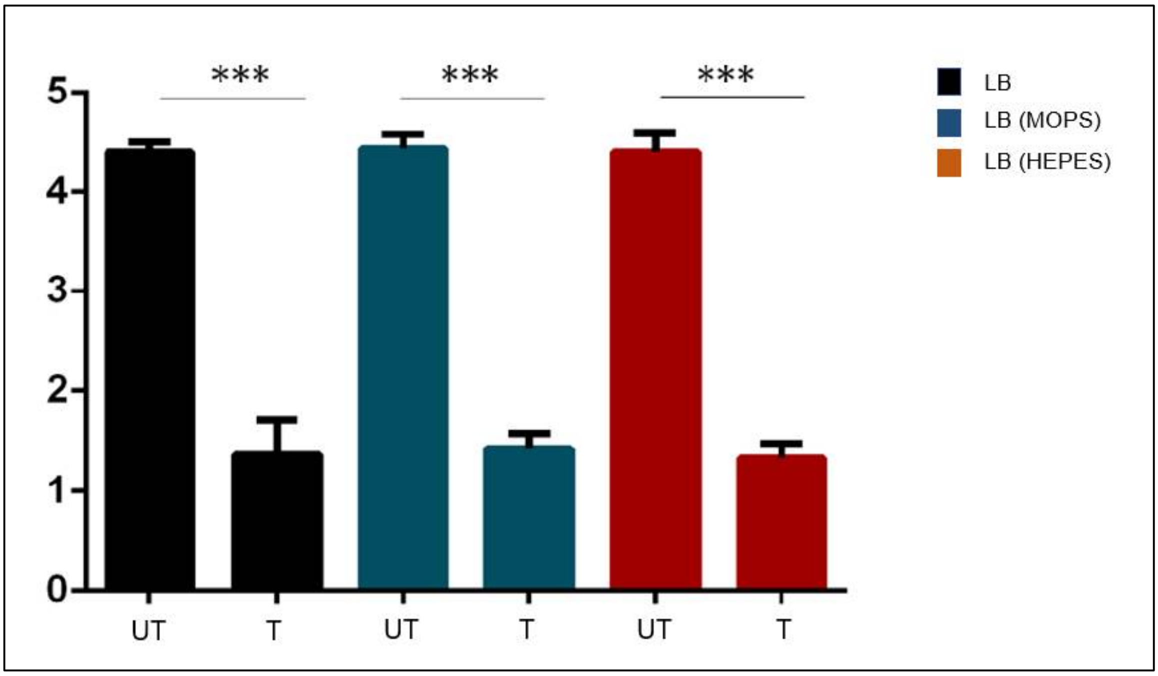

Supplementary Figure S3. *Salmonella* motility in buffered media. Bars represent bacterial mobility in semisolid LB media (black bars) or with MOPS (green bars) and HEPES (red bars) pH buffers. Motility of *Salmonella* decreases in the media treated (T) with 0.5 mg/mL of *Hibiscus sabdariffa* L. extract compared with untreated media (UT). The trial was conducted in triplicate and the bar represents the standard deviation. P > 0.05 was considered as a significant difference.

Supplementary Table S1. Composition of the mobile phase used for the fractionation of the total extract of *H. sabdariffa* L. by column chromatography and biological activity.

Mobile phase	Vol. (mL)	Fractions	Inhibition of *Salmonella* motility
Hexane	200	1-4	Negative
Hexane-ethyl acetate 9:1	100		
Hexane-ethyl acetate 8:2	100		
Hexane-ethyl acetate 7:3	100	5-6	Negative
Hexane-ethyl acetate 6:4	100		
Hexane-ethyl acetate 5:5	100		
Hexane-ethyl acetate 3:7	100	7-10 (“Fraction A”)	Positive
Hexane-ethyl acetate 1:9	100		
Ethyl acetate-methanol 9:1	100		
Ethyl acetate-methanol 8:2	100		
Ethyl acetate-methanol 7:3	100	11-14	Negative
Ethyl acetate-methanol 6:4	100		
Ethyl acetate-methanol 5:5	100		
Ethyl acetate-methanol 3:7	100		
Methanol	500		ND*

*. Not determined

Supplementary Table S2. Composition of the mobile phase used for the fractionation of the "Fraction A" by column chromatography and biological activity.

Mobile phase	Vol. (mL)	Fractions	Inhibition of *Salmonella* motility
Hexane	100	1-8	Negative
Hexane-ethyl acetate 9:1	100		
Hexane-ethyl acetate 8:2	200		
Hexane-ethyl acetate 7:3	100	9-13	Negative
Hexane-ethyl acetate 6:4	100		
Hexane-ethyl acetate 5:5	100		
Hexane-ethyl acetate 3:7	100	14-17 (“Fraction B”)	Positive
Hexane-ethyl acetate 1:9	100		
Ethyl acetate-methanol 9:1	200	18-46	Negative
Ethyl acetate-methanol 8:2	200		
Ethyl acetate-methanol 7:3	100	47-54	Negative
Ethyl acetate-methanol 6:4	100		
Ethyl acetate-methanol 5:5	100	55-69	Negative
Ethyl acetate-methanol 3:7	100		
Methanol	500		ND*

*. Not determined

Supplementary Table S3. Composition of the mobile phase used for the fractionation of "Fraction B" by column chromatography and biological activity.

**Mobile phase**	**Vol. (mL)**	**Fractions**	**Inhibition of *Salmonella* motility**
Hexane	100	1-2	Negative
Hexane-ethyl acetate 5:5	100		
Hexane-ethyl acetate 1:9	100	3-5 (“Fraction C”)	Positive
Ethyl acetate-methanol 8:2	100		
Ethyl acetate-methanol 6:4	100	6-9	Negative
Ethyl acetate-methanol 3:7	100		
Ethyl acetate-methanol 2:8	100		
Methanol	300	10-15	Negative
